# Machine learning classification of active viewing of pain and non-pain images using EEG does not exceed chance in external validation samples

**DOI:** 10.3758/s13415-025-01268-2

**Published:** 2025-02-18

**Authors:** Tyler Mari, S. Hasan Ali, Lucrezia Pacinotti, Sarah Powsey, Nicholas Fallon

**Affiliations:** Department of Psychology, Institute of Population Health, Faculty of Health and Life Sciences, University of Liverpool, Bedford Street South, Liverpool, L69 7ZA UK

**Keywords:** Empathy, Electroencephalography, Event-related potential, Random forest, Faces

## Abstract

Previous research has demonstrated that machine learning (ML) could not effectively decode passive observation of neutral versus pain photographs by using electroencephalogram (EEG) data. Consequently, the present study explored whether active viewing, i.e., requiring participant engagement in a task, of neutral and pain stimuli improves ML performance. Random forest (RF) models were trained on cortical event-related potentials (ERPs) during a two-alternative forced choice paradigm, whereby participants determined the presence or absence of pain in photographs of facial expressions and action scenes. Sixty-two participants were recruited for the model development sample. Moreover, a within-subject temporal validation sample was collected, consisting of 27 subjects. In line with our previous research, three RF models were developed to classify images into faces and scenes, neutral and pain scenes, and neutral and pain expressions. The results demonstrated that the RF successfully classified discrete categories of visual stimuli (faces and scenes) with accuracies of 78% and 66% on cross-validation and external validation, respectively. However, despite promising cross-validation results of 61% and 67% for the classification of neutral and pain scenes and neutral and pain faces, respectively, the RF models failed to exceed chance performance on the external validation dataset on both empathy classification attempts. These results align with previous research, highlighting the challenges of classifying complex states, such as pain empathy using ERPs. Moreover, the results suggest that active observation fails to enhance ML performance beyond previous passive studies. Future research should prioritise improving model performance to obtain levels exceeding chance, which would demonstrate increased utility.

## Introduction

Pain empathy is the capacity to identify, recognise, and resonate with another individual’s pain and is an imperative function for avoiding harm and promoting prosocial behaviour (Decety et al., 2016; Hein et al., 2010; Zhou et al., 2020). Pain empathy can be elicited experimentally by using photographs of painful scenarios, such as depicting actual or implied physical harm, or through images of painful expressions, e.g., grimacing (Coll, 2018; Jauniaux et al., 2019; Mari et al., 2023c). Understanding psychological and neurobiological responses during pain observation is imperative for clinical, physiological, and societal research (Decety & Jackson, 2004; Lamm et al., 2011; Singer et al., 2004; Singer & Lamm, 2009). For example, further understanding the neurobiology of pain empathy may help to improve areas, such as medical education, where bias and corresponding changes to empathy can hinder accurate pain assessment (Hoffman et al., 2016; Preusche & Lamm, 2016).

Previous research has consistently demonstrated differences in neuronal activation during pain observation using neuroimaging methods, such as electroencephalography (EEG) and functional magnetic resonance imaging (fMRI) (Coll, 2018; Fallon et al., 2020). Electroencephalogram research exploring differences in event-related potentials (ERPs) has demonstrated that pain observation enhanced P3 and late positive potential (LPP) components, with the maximal effect recorded over central-parietal electrodes (Coll, 2018). Recently research by our lab further demonstrated these effects, revealing an enhanced LPP over central-parietal regions during passive observation of pain scenes compared to matched non-pain neutral scenarios (Mari et al., 2023c). Moreover, we also identified augmented P3 amplitudes over central-parietal electrodes while observing pain faces (expressions) when compared to matched neutral expressions. Altered ERP responses to observation of painful scenes have also been observed in both healthy people and a chronic pain population (Fallon et al., 2015a).

Augmented ERP waveforms during observation of painful, compared to neutral, images could provide sufficient discrimination to develop machine learning (ML) algorithms for the classification of pain empathy from neural responses alone. The combination of ML and EEG has already demonstrated promise across several domains, including classifying neural responses to visual stimuli (Kaneshiro et al., 2015; Stewart et al., 2014; Zheng et al., 2020), subjective pain intensity (Mari et al., 2022, 2023a), and pain phenotypes (Levitt et al., 2020; Mari et al., 2022; Ta Dinh et al., 2019) to name but a few. Despite the potential applications of a pain empathy prediction algorithm, there is currently a paucity of research using ML to decode neural responses from EEG data.

Recent research using fMRI and ML has attempted to classify empathic responses to vicarious pain scene images and facial expressions (Zhou et al., 2020). They demonstrated that neural responses during observation of neutral and painful scene images could be classified with a cross-validation accuracy of 88%. Moreover, the results demonstrated that neutral and painful expressions could be classified with a cross-validated accuracy of 80%. In addition, previous research from our lab aimed to develop the first approach for using ML and EEG to classify neural responses during passive observation of pain scenes and faces compared with perceptually matched neutral stimuli (Mari et al., 2023c). Despite identifying differences in the ERP components at the group level, random forest (RF) models of ERPs from single trials were unable to classify neutral or painful images of either scene or face photographs with accuracies greater than the chance level on external validation samples.

One potential explanation for the inability of ML to classify pain empathy using neural responses is the implementation of a passive viewing paradigm. Research has demonstrated that ERPs are attenuated during passive viewing compared with active viewing (Bennington & Polich, 1999). The previous research identified that P300 amplitudes were larger during active viewing in response to both auditory and visual stimuli compared with passive viewing (Bennington & Polich, 1999). Moreover, research has demonstrated that other components, such as the LPP, are altered by levels of attention and engagement (Dunning & Hajcak, 2009; Hajcak et al., 2013; Kam et al., 2014). Given the low signal-to-noise ratio of single-trial EEG, any additional attenuation of the ERPs is likely to hinder model performance (Blankertz et al., 2011). Therefore, employing an active-viewing paradigm, which requires engagement from participants in the form of a response or feedback may increase the robustness of the ERP responses, improving classification performance.

Large differences in specific ERP components have been successfully used to discriminate discrete categories of visual stimuli using ML, as evidenced by our previous findings (Mari et al., 2023c). We found that ML could accurately classify scene and face images (regardless of the pain component), with accuracies of 75%, 64%, and 69% for cross-validation, cross-subject external validation, and within-subject external validation, respectively. The location of the features suggests that both the N170 component and vertex positive potential likely contributed to the classification performance. Research has demonstrated that the N170 component is maximally responsive during the observation of faces and is absent or diminished in response to non-face stimulus categories (Bentin et al., 1996; Bötzel et al., 1995; Mari et al., 2023c; Soto et al., 2018). Moreover, the N170 component has been established as the most important electrophysiological index of face processing (Hinojosa et al., 2015). In contrast, the vertex positive potential is a positive potential across frontal-central regions and is observed during presentation of face stimuli (Bentin et al., 1996; Bötzel et al., 1995). The N170 component also appears to provide discriminative ability at the single-trial level, which is imperative for ML analyses. For example, Tian & colleagues (2018) found that single-trial N170 waveforms could be used to effectively classify face emotions. Taken together, the evidence suggests that the N170 component is important for the classification of face and non-face stimuli at both single-trial and average levels.

The present study was designed to develop and externally validate (within-subjects) a pain empathy classification algorithm using EEG data. We developed an active-viewing paradigm to elicit enhanced ERP signals, which may contribute to improved classification performance. Initially, we aimed to develop a RF model to classify scene or face images (regardless of pain component) using EEG data to replicate our previous findings (Mari et al., 2023c). Random forest models were selected, because they have demonstrated reasonable classification accuracy and calibration in previous literature (Mari et al., 2023a, b; Vijayakumar et al., 2017). Additionally, the models are generally robust to overfitting (Bentéjac et al., 2021; Breiman, 2001; Dong et al., 2020; Fernández-Delgado et al., 2014). Given the risk of overfitting in the ML literature (Mari et al., 2022), RF models were deemed optimal owing to their robustness and potential effectiveness. Subsequently, we aimed to classify neutral and painful scenes and neutral and painful faces using two additional RF models. All the developed models were externally validated using a within-subjects temporal external validation dataset, consisting of participants who completed the study a second time at a later date. We predicted that the RF model would classify the active observation of visual stimuli with accuracies greater than chance level (≈50%) on the external validation dataset for each of the three classification tasks: 1) faces and scenes, 2) neutral and pain scenes, and 3) neutral and pain faces (expressions).

## Methods

### Participants

A total of 62 participants (37 females, 12 left-handed) aged between 19 and 68 years (mean = 28.60 years, standard deviation [SD] = 11.91) were recruited for the model development sample using an opportunity sampling method. Participants were invited to complete the experiment a second time after a minimum of 6 weeks from their first session (mean = 61.93 days, SD = 11.82) to create a temporal within-subject external validation sample for the ML analysis. A total of 27 participants (11 females, 5 left-handed) aged between 19 and 64 years (mean = 28.56 years, SD = 11.63) completed the experiment for a second time. Participants were required to be at least 18 years, have normal, or corrected-to-normal vision, have no acute pain at the time of participating, have no history of chronic pain, and no neurological conditions. The study received ethical approval from the University of Liverpool Health and Life Sciences Research Ethics Committee. Fully informed written consent was obtained from participants before each session, and all methods were conducted in compliance with the Declaration of Helsinki. Participants were reimbursed £40 for each session for travel and time expenses. The raw data are freely available through the Open Science Framework (https://osf.io/2e8zk/).

### Materials

#### Pain faces

All visual stimuli employed in this study were identical to our previously published research (Mari et al., 2023c). A 2 x 2 factorial design was implemented in this study. The conditions were faces (expressions) and scenes, which both consisted of neutral and vicarious pain. All images were displayed in the centre of the screen. Both the pain expression and matched neutral images were chosen from the Delaware Pain Database (Mende-Siedlecki et al., 2020). A total of 56 images were selected (28 pain expressions and 28 matched neutral images) using several criteria. First, the images were selected to approximately match the ethnicity and gender distribution of the United Kingdom (Office for National Statistics, 2021), with a total of 22 White subjects (80%), consisting of equal males and females; three Black subjects (10%), consisting of one male and two females; and three Asian subjects (10%), including two males and one female. Within each of the individual ethnicity and gender categories, the images that received the highest pain rating and were listed as pain being the dominant emotion were selected. The 28 neutral images were chosen as the matched version of the pain expression, which contained the same subject. The images were approximately 1382 x 925 pixels in size.

#### Pain scenes

**Fig. 1:**
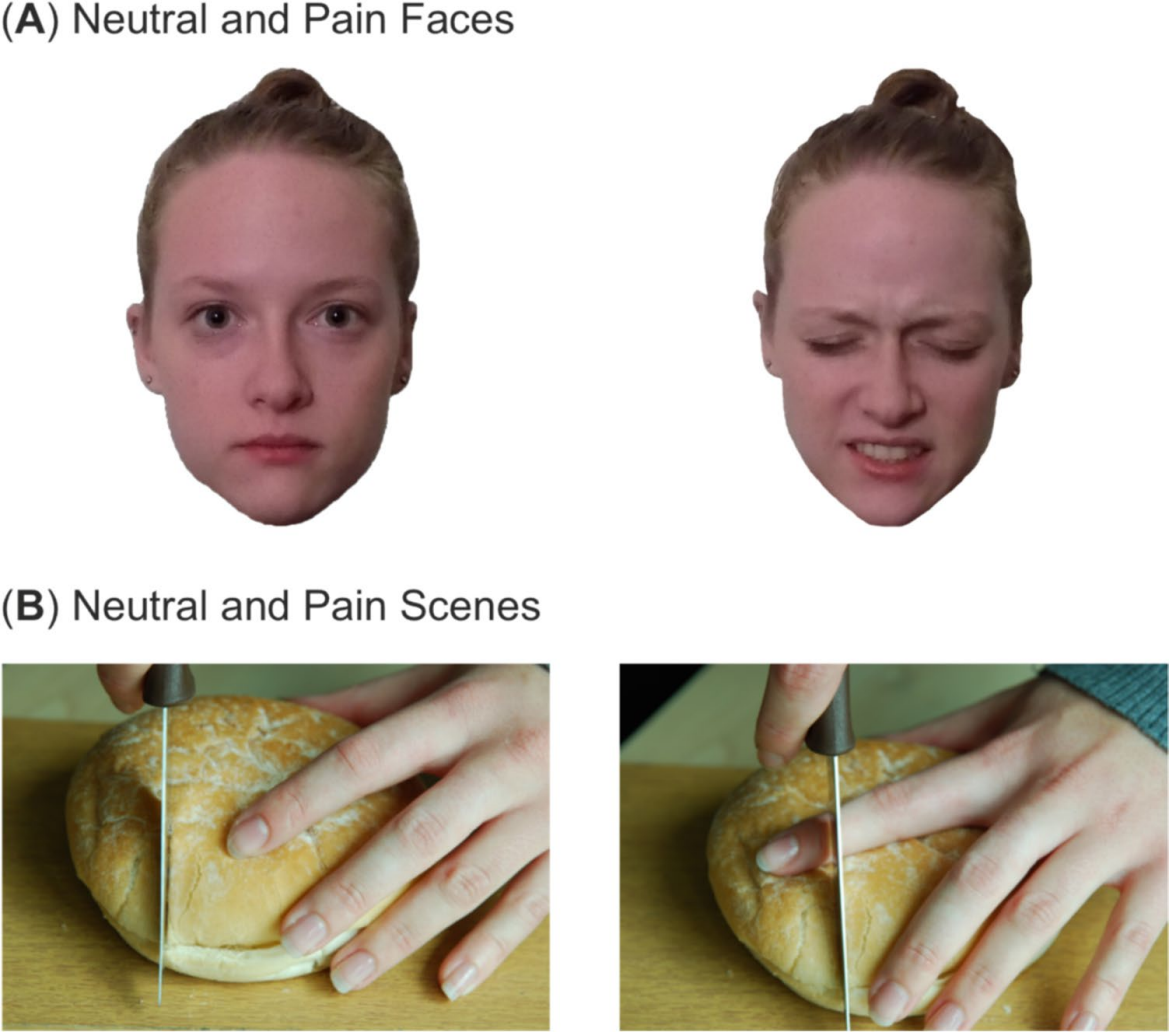
Examples of neutral and pain stimuli conditions. **A** Neutral and pain face conditions from the Delaware Pain Database (Mende-Siedlecki et al., 2020). **B** Neutral and pain scene stimuli examples

In addition to the pain faces, still photograph images of action scenes depicting pain or matched non-pain scenarios were presented in this study. A total of 28 images, which depicted the hands or feet in situations that elicit pain, were used in this study. For example, a photograph of a knife cutting bread in a manner that would endanger the finger (e.g., positioned under the knife). Additionally, 28 matched neutral scene photographs, replicating the action scene without endangering the subject were used (e.g., knife cutting bread with no danger to finger). The ethnicity and gender distribution of subjects used for the scene images matched that of the face images. The pain scene images are comparable to previously published research (Fallon et al., 2015a, b; Fan & Han, 2008). The pain scene images were 774 x 518 pixels in size. Figure 1 provides examples of the photographs of the face and scene conditions used in this study.

### Procedure

Participants attended the EEG laboratory at the University of Liverpool between July 2023 and October 2023. Initially, the EEG cap was fitted, and the participants were seated inside the Faraday cage approximately 1 m away from a 23-inch 1080p LCD monitor. The experimenter verbally explained that participants were required to attend to the images and evaluate whether pain was present in the image (e.g., whether the image was neutral or painful). The participants were instructed to respond by using the left or right arrow buttons on the keyboard, with the left arrow indicating the absence of pain and the right arrow indicating the presence of pain. Participants were also informed that they would be provided with an accuracy score at the end of the block, which provided the percentage of correctly identified trials to promote engagement. Finally, the participants were instructed to minimise movement during the trials.

The experiment implemented a two-alternative force choice paradigm, whereby participants were required to determine the presence or absence of pain. Each trial began with a blank grey screen, comprising a 2-s rest interval. Following the rest period, a colour photograph was randomly displayed for 1 second. Subsequently, the screen was cleared, and a 1-s rest interval was completed. Participants were then required to report whether the image contained pain. The question “Was pain present in the image?” was presented at the top of the screen, and two arrows were presented in the middle of the screen, which pointed in opposite directions to remind participants of which keys to select. The left arrow was labelled “no,” which indicated pain was not present in the image, whilst the right arrow was labelled yes, indicating that pain was present in the image. Here, an infinite wait time was implemented, and the trial did not progress until the participant had selected either the left or the right key. Following this, the screen was cleared, and the next trial began with the 2-s rest period. This was repeated until all 112 images had been presented. On completion of each block, participants were informed that they had completed the block and were provided their accuracy score as a percentage.

Each block consisted of 112 trials, with 28 stimuli for each of the four conditions of the study. A total of three blocks were conducted, with a total of 336 trials for the entire experiment. The images were the same across all three blocks but were randomly presented. The duration of the blocks was approximately 8 min, and each block was separated by approximately 15 min.

Once the subject had completed all the blocks, they were required to complete a subjective rating block. The participants were informed that they were required to rate their perceived pain intensity of each of the 112 images on a 0–100 scale: 0 reflected no pain, and 100 reflected extreme pain. The rating scale had vertical bars denoting increments of 10. Each image was presented for an infinite period and was positioned directly above the rating scale. Participants responded by clicking the scale in the desired position using the mouse in their right hand. The image presentation was randomised and after the participant had successfully rated an image, the screen was cleared, and the subsequent photograph was presented 100 ms later. Finally, participants completed the pain catastrophising scale (PCS) (Sullivan et al., 1995). The PCS is a self-report questionnaire, comprised of 13 items, that assess an exaggerated negative mental set brought to bear during actual or anticipated pain experience and includes items, such as “I can’t stop thinking about how much it hurts” and “There is nothing I can do to reduce the intensity of my pain.” Following completion of the PCS, participants were debriefed and compensated for their participation.

### EEG acquisition and analysis

Continuous EEG recordings were acquired by using a 129-channel EEG system (Electrical Geodesic Inc., EGI, Magstim EGI, Eugene, OR) and a sponge-based Geodesic sensory net. The correct net position was achieved with respect to three anatomical landmarks two preauricular points and the nasion. Electrode-to-skin impedances were maintained below 50 kΩ for the duration of the experiment, with additional saline solution applied as required in-between blocks. The sampling rate was set at 1000 Hz, and a recording bandpass filter between 0.001 and 200 Hz was applied. Electrode Cz was used as the reference electrode.

In line with previous research conducted by our lab (Mari et al., 2023c), the EEG data were pre-processed by using the Harvard Automated Processing Pipeline for Electroencephalography (HAPPE version 3) (Gabard-Durnam et al., 2018). HAPPE is an automated software for EEG pre-processing written in MATLAB (Gabard-Durnam et al., 2018). The software consists of the core processing steps for EEG, including data filtering (low-pass/high-pass filters), re-referencing, epoching, baseline correction, bad channel rejection, automated segment rejection, and bad channel interpolation (Gabard-Durnam et al., 2018).

The data were filtered between 0.1 and 45 Hz by using high-pass and low-pass filters, and were referenced by using the common average approach (Lehmann, 1987). The data were down-sampled to 500 Hz. Bad channels, which did not contain usable brain data due to artefacts, were identified and interpolated, followed by artefact correction using the wavelet thresholding approach with a soft margin to remove artefact data and isolate neural data. Bad channel detection identifies channels using several criteria, including the identification of flatline channels, and rejection of channels based on the correlation coefficient with other channels (Gabard-Durnam et al., 2018; Monachino et al., 2022). In addition, wavelet artefact correction is a computationally efficient method that locates artefacts using frequency and time localisation and efficiently removes the artefacts without distorting the original brain signal (Gabard-Durnam et al., 2018; Monachino et al., 2022). The continuous data were then segmented into epochs of −200 ms to 800 ms relative to stimulus onset and were baseline corrected (−200 ms to 0 ms). Following this, bad channel identification and interpolation within the epochs were conducted. Conducting bad channel identification within segments allows the user to minimise the number of epochs removed during trial rejection, whilst maximising the correction of artefacts within each segment (Gabard-Durnam et al., 2018). Within-epoch interpolation was conducted to maximise the number of observations, as larger datasets are less prone to overfitting during ML model development (Vabalas et al., 2019). Finally, automatic trial rejection was implemented by using both the segment amplitude and similarity criteria. Thresholds for the minimum and maximum amplitude were set at −150 mV and 150 mV in line with HAPPE recommendations (Gabard-Durnam et al., 2018).

Following trial rejection, the number of trials (mean ± SD) remaining in the model development sample was 60.16 ± 6.45 (72% of trials remaining) for neutral scenes, 60.31 ± 6.94 (72%) for pain scenes, 62.32 ± 6.49 (74%) for neutral faces, and 61.56 ± 7.18 (73%) for pain faces. Similar results were obtained for the temporal within-subjects external validation sample with 63.30 ± 5.62 (75%) trials remaining for neutral scenes, 61.15 ± 6.63 (73%) remaining for pain scenes, 63.96 ± 6.90 (76%) remaining for neutral faces, and 62.74 ± 7.09 (75%) remaining for pain faces. The ERPs were analysed using MATLAB 2022b (The MathWorks, Inc., Natick, MA) and EEGLAB 2022.0 (Delorme & Makeig, 2004). The false discovery rate (FDR) method was implemented to correct for multiple comparisons. A minimum window width of 10 ms was implemented to assess the significant differences between the ERP waveforms.

### Machine learning procedure

The ML processing procedure was conducted following the approach of our previously published research (Mari et al., 2023c). Both the model development and external validation dataset were processed separately to prevent data leakage, which could artificially inflate external validation performance (Luo et al., 2016). First, candidate features were calculated by using the single-trial ERP waveforms between 0 and 800 ms relative to stimulus onset. The features primarily consisted of descriptive statistics of the waveform and are consistent with our previous work (see Mari et al., 2023a, b, c) as well as comparable to further work conducted by our lab and externally (Anuragi & Sisodia, 2020; Mari et al., 2023a, b; Sai et al., 2019). The features include measures of central tendency, such as the mean, dispersion (e.g., standard deviation), distribution features, such as skewness, and entropy measures (e.g., Shannon Entropy). The features were calculated by using standard MATLAB functions, where possible, for all 129 electrodes.

Following this, outlier interpolation was implemented due to signal noise and variability issues associated with single-trial EEG (Faisal et al., 2008; Kaplan et al., 2005; Marathe et al., 2014), which can often hinder ML performance (Maniruzzaman et al., 2018). Subsequently, feature values exceeding 3 median absolute deviations were linearly interpolated using the *filloutliers* function in MATLAB 2022b (The MathWorks, Inc.). In the model development sample, a total of 4.74 ± 0.33% of trials were interpolated, whereas for the within-subject external validation dataset, a total of 4.94 ± 0.32% of trials were interpolated. Data interpolation was conducted to maximise the dataset size, as small samples are prone to overfitting (Vabalas et al., 2019).

After the data processing had been completed in MATLAB, the data were analysed by using Python and Scikit-learn (Pedregosa et al., 2011). Initially, the random seed was defined (seed = 123) and the features were scaled to between 0 and 1. Subsequently, feature selection using F-tests was conducted to rank the features in terms of importance. Following this, a custom sequential selection feature selection algorithm was implemented. Here, a RF model was trained by using one feature initially with no hyperparameter optimisation. Random forest models were selected, because they generally perform well with minimal hyperparameter optimisation, are robust to overfitting, and provide good performance on real-world data (Bentéjac et al., 2021; Breiman, 2001; Dong et al., 2020; Fernández-Delgado et al., 2014). In addition, our previous research demonstrated that RF models outperformed other common models, such as support vector machines (Mari et al., 2023a), and provided good calibration performance both within and across subjects (Mari et al., 2023c). Features were then sequentially added to the model to identify the best feature set. The feature set was selected as the model that achieved the best cross-validation accuracy. This process was conducted for all three classification attempts (faces – scenes, scenes: neutral and pain, and faces: neutral and pain). For all classifications, a maximum of 100 features were tested to limit model complexity and reduce the risk of overfitting. We limited the number of potential features to 100 to minimise the risk of overfitting by maximising the ratio of observations and predictors. Estimates suggest that the number of observations should be between 50 and 200 times the number of predictors (Riley et al., 2020; Van Der Ploeg et al., 2014). Consequently, by setting the maximum features at 100, we ensured that the ratio of observations to features was at least 150:1 for the faces and scenes classification, and approximately 75:1 for both pain empathy classification tasks, which is in line with the recommendations (Riley et al., 2020; Van Der Ploeg et al., 2014).

Once the optimal feature set had been identified, a final RF model was developed for each of the three classification tasks. A random search approach, with a maximum of 10,000 iterations, was conducted. Cross-validation was achieved using stratified k-fold validation with accuracy as the scoring function. Following the identification of the optimal hyperparameters, the model was refitted to the entire training set, which resulted in the final model (for each classification attempt) that was subsequently evaluated using the external validation dataset.

#### Model evaluation: discrimination and calibration

To evaluate the predictive capability of each model, we assessed several performance metrics for both the cross-validation and external validation. The metrics implemented in this study have been assessed in our previously published work (Mari et al., 2023a, c). The key discrimination metrics implemented were accuracy and area under the receiver operating characteristics curve (AUC), with additional metrics of precision, recall, F1 score, and Brier score also reported. Comprehensive overviews of these performance metrics are available elsewhere (Alba et al., 2017; Mari et al., 2022; Sokolova & Lapalme, 2009). To calculate model performance on the external validation dataset, the model was evaluated on the data from each subject individually and was averaged to obtain the sample performance.

In addition, we assessed calibration for models that statistically exceed chance performance (Alba et al., 2017). Calibration evaluates the agreement between the model’s predicted probability of an event and the observed reference value (Alba et al., 2017; Luo et al., 2016; Van Calster et al., 2019). Calibration curves, which display the true probability of the event on the y-axis and predicted probability on the x-axis, are the preferred method for evaluating calibration (Moons et al., 2015; Van Calster et al., 2016). The probabilities were segmented into bins (20 in the present study) with equal intervals between 0 and 1 (Huang et al., 2020). Perfect calibration occurs when the predicted and observed probabilities are the same and are represented by a 45° line on the calibration plot.

#### Statistical thresholding

To evaluate whether ML performance on the within-subjects external validation sample statistically exceeded chance, statistical thresholding using the approach proposed by Combrisson and Jerbi (2015) was conducted. For a binary classification task with an infinite sample size, the theoretical chance level is 50%. However, sample sizes are finite and often small in neuroscientific research, resulting in variable chance levels. Therefore, statistical analysis is required to determine whether model performance exceeds chance levels based on finite trials and sample sizes. Whilst permutation testing is an effective approach, the method is computationally expensive, with the computational cost increasing with sample size and statistical significance threshold (Combrisson & Jerbi, 2015). Therefore, we implemented the binomial cumulative distribution approach, which provides comparable results to permutation testing and is significantly less computationally expensive (Combrisson & Jerbi, 2015). Statistical thresholds can be calculated by using the following MATLAB function:

$$Statistical\; Threshold=binoinv\left(1- a,n,\frac{1}{c}\right)* \frac{100}{n}$$

Where α is the significance threshold, *n* is the number of trials for each subject, and *c* is the number of classes. For example, for a binary classification task and a sample with *n* = 200, the model accuracy must exceed 56%, 58%, and 61% to be significant at the 0.05, 0.01, and 0.001 levels (Combrisson & Jerbi, 2015). If the model accuracy exceeds the threshold value, then the model performance is significantly greater than the chance level.

One hundred data samples are required to achieve results comparable to permutation testing (Combrisson & Jerbi, 2015). Of the 27 subjects of the external validation dataset, 26 had a minimum of 100 trials and were suitable for the use of binomial testing. One subject did not meet the required 100 trials, with only 92 trials for the neutral and pain face classification. Despite this, statistical thresholding was still conducted on this subject to provide an approximation of the model performance. In all classification attempts, *p* = 0.05 was used as the threshold for significant differences. The average chance level for a two-class classification task, with *p* = .05, was determined as 55.22 ± 0.29%, 56.46 ± 0.42%, and 57.33 ± 0.47% for faces – scenes, scenes: neutral – pain, and faces: neutral – pain, respectively. Finally, we assessed whether, across the sample, the accuracy exceeded chance levels. To evaluate this, paired samples *t*-tests were conducted to test whether model accuracy significantly exceeded the chance levels across the sample. All methods were conducted following our previously published research (Mari et al., 2023c).

## Results

### Self-report ratings

The descriptive statistics of the average self-report ratings for the four image conditions and samples are reported in Table 1. First, a 2 x 2 repeated measures ANOVA was conducted using IBM SPSS 29 (IBM Corp., Armonk, NY) to assess differences in self-report pain ratings for the different image categories in the model development sample. The results demonstrated a significant main effect of pain condition on subjective pain intensity ratings (F (1,61) = 1000.41, *p* < .001, η_p_^2^ = .943), with the pain images receiving greater pain ratings than the neutral images. There was also a significant main effect of image type on the participant’s subjective pain intensity ratings (F (1,61) = 28.73, *p* < .001, η_p_^2^ = .320), with the scene images receiving higher pain ratings than the face images. The results also demonstrated a significant interaction between pain condition and image type (F (1,61) = 34.94, *p* < .001, η_p_^2^ = .364). Post-hoc paired samples *t*-tests revealed that the pain scene images received significantly higher pain ratings when compared to the pain faces condition (t (61) = 5.85, *p* < .001, d = .743, 95% CI = [.459, 1.022]). In addition, both the pain scenes and pain faces received significantly higher pain ratings than the neutral scenes (t (61) = 34.71, *p* < .001, d = 4.408, 95% CI = [3.587, 5.225]) and neutral faces (t (61) = 23.36, *p* < .001, d = 2.93, 95% CI = [2.383, 3.546]), respectively. Finally, there was no significant difference in perceived pain intensity ratings between the neutral faces and scenes (t (61) = 0.15, *p* = .884, d = 0.19, 95% CI = [−.230, .267]).

**Table 1:** Mean ± SD of perceived pain intensity for each condition for both model development sample and within-subject external validation sample

Sample	Neutral Scenes	Neutral Faces	Pain Scenes	Pain Faces
Development sample (n = 62)	2.56 ± 2.92	2.49 ± 4.55	62.91 ± 14.12	52.53 ± 17.38
External validation sample (n = 27)	1.29 ± 1.71	1.49 ± 2.71	64.47 ± 14.48	57.27 ± 19.31

### Machine learning

**Fig. 2:**
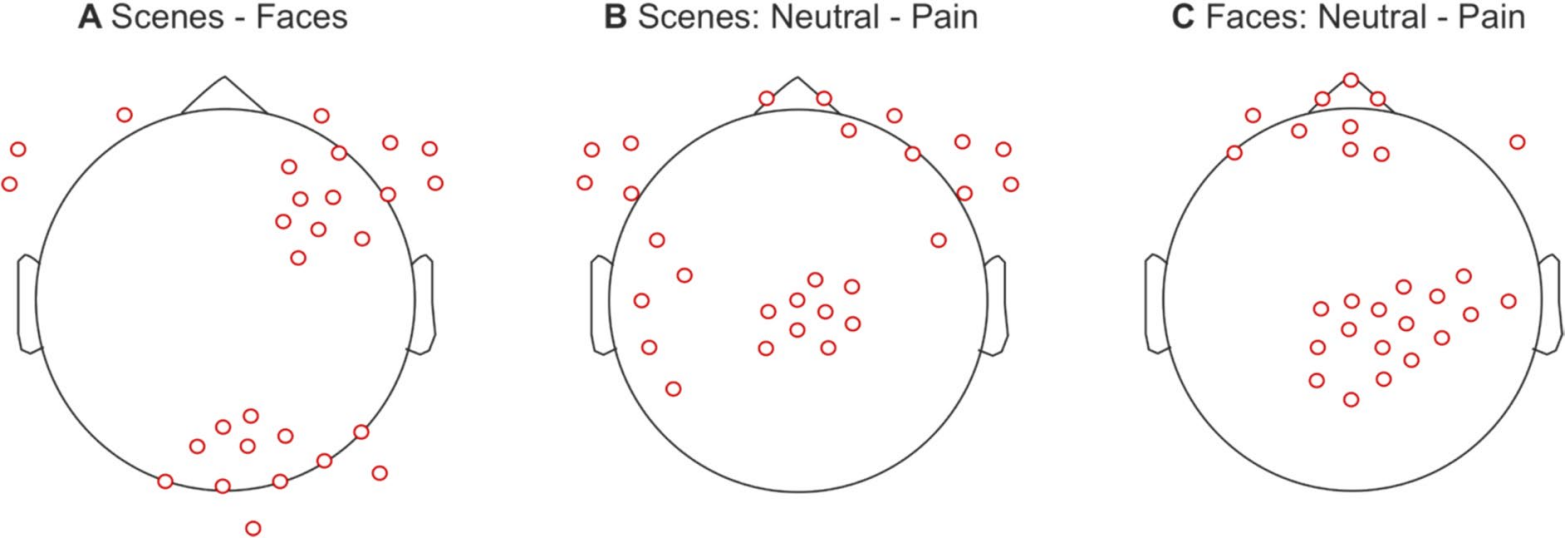
Scalp locations of the features used to develop the ML model for each classification task. Scenes—faces (**A**), scenes: neutral—pain (**B**), and faces: neutral—pain (**C**)

From the feature selection process, a total of 98, 89, and 99 features were deemed optimal for each classification task, namely scenes – faces, neutral and pain scenes, and neutral and pain faces, respectively. The scalp locations for each of the different classification attempts are presented in Fig. 2. For the scenes–faces classification, the features are predominantly located over right-frontal electrodes and right and central occipital regions. The features for the neural and pain scene classification are located predominantly over the vertex as well as left and right frontal regions, extending to parietal and temporal regions on the left side. For the neutral and pain face photographs some features are located over frontal regions extending from central to left-sided electrodes. However, most features are located in a cluster over the central and right parietal regions. Finally, the number of observations for each classification attempt and sample are reported in Table 2.

**Table 2:** Number of trials per condition and sample used in the ML analysis

	Scenes		Faces		
Sample	Neutral	Pain	Neutral	Pain	Total
Development	3,730	3,739	3,864	3,817	15,150
Within-subject	1,709	1,651	1,727	1,694	6,781
Total	5,439	5,390	5,591	5,511	21,931

### Faces – scenes classification

**Fig. 3:**
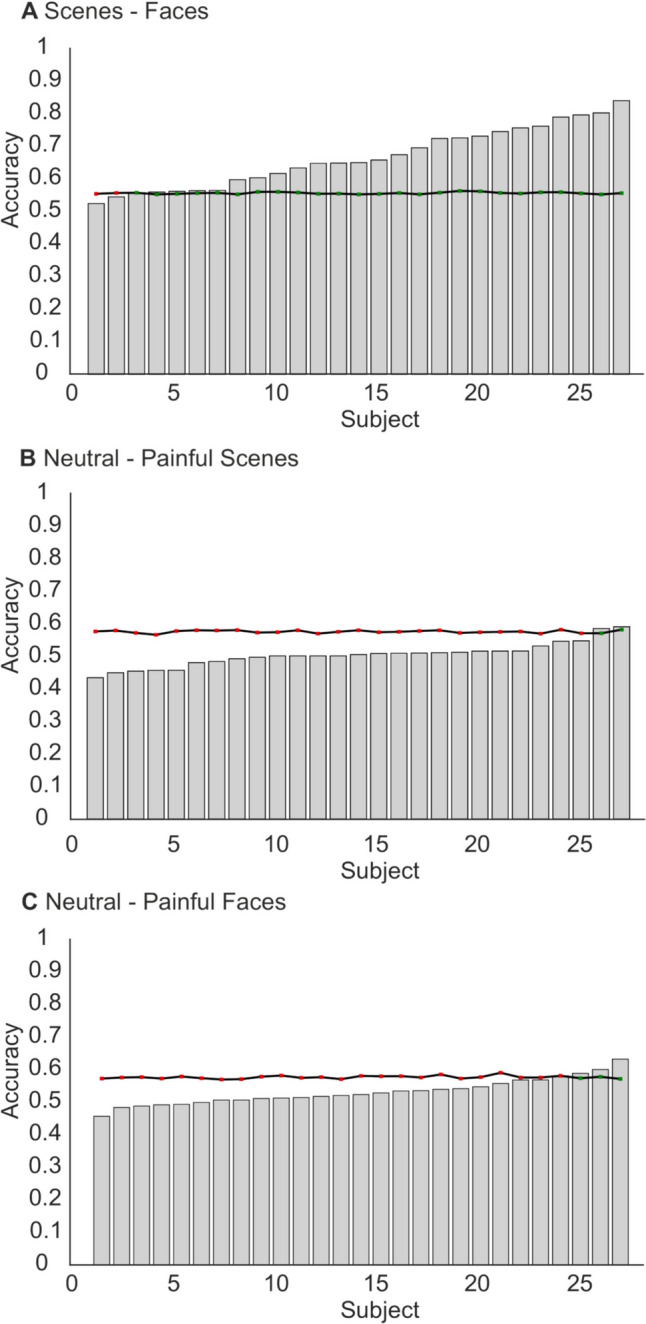
Model accuracies and chance threshold for individual subjects from the within-subjects external validation dataset for the Scenes – Faces classification (**A**), Neutral – pain scenes classification (**B**), and Neutral – pain – faces classification (**C**). The black lines denote the significance threshold for chance classification performance at *p* = .05 Red squares represent performance that did not exceed chance levels. Green squares denote subjects where the model significantly exceeded chance performance

The results for the faces – scenes classification, as well as the optimal hyperparameters, are reported in Table 3. The average sample results demonstrated that the RF model achieved a cross-validation accuracy (± SD) of 0.7789 ± 0.0460 and an AUC of 0.8557 ± 0.0461. This indicates that the model correctly identified whether the participant was viewing the face or scene category on 78% of the training set trials. For the within-subjects external validation sample, the model achieved a mean sample accuracy of 0.6609 ± 0.912 and an AUC of 0.7424 ± 0.0961. Paired samples *t*-tests demonstrated that the average subject performance was significantly greater than chance levels at *p* = .05 (t (26) = 6.23, *p* < .001, d = 1.198, 95% CI = [.694, 1.689]) and *p* = .001 thresholds (t (26) = 3.66, *p* = .001, d = .703, 95% CI = [.268, 1.088]). For the individual subject performance, the model demonstrated greater accuracy than chance, with the model demonstrating performance that exceeded chance levels (*p* = .05) for 24 of 27 subjects. Figure 3 displays the accuracies and chance thresholds for individual subjects in the external validation set on all three classification attempts.

**Table 3:** Average sample performance metrics and optimal hyperparameters for the scenes-faces classification

	Cross-Validation		Within-Subject Validation	
Metric	Mean	SD	Mean	SD
Accuracy	0.7789	0.0460	0.6609	0.0912
AUC	0.8557	0.0461	0.7424	0.0961
Brier Score	0.1538	0.0213	0.2069	0.0339
F1 Score	0.8159	0.0302	0.7410	0.0555
Precision	0.7126	0.0495	0.6119	0.0719
Recall	0.9577	0.0161	0.9486	0.0541

**Optimal hyperparameters**: Number of estimators = 960, Maximum depth = 93, Minimum samples to split = 23, Minimum samples at leaf = 2, Maximum features = log2, Bootstrap = True

#### Calibration

**Fig. 4:**
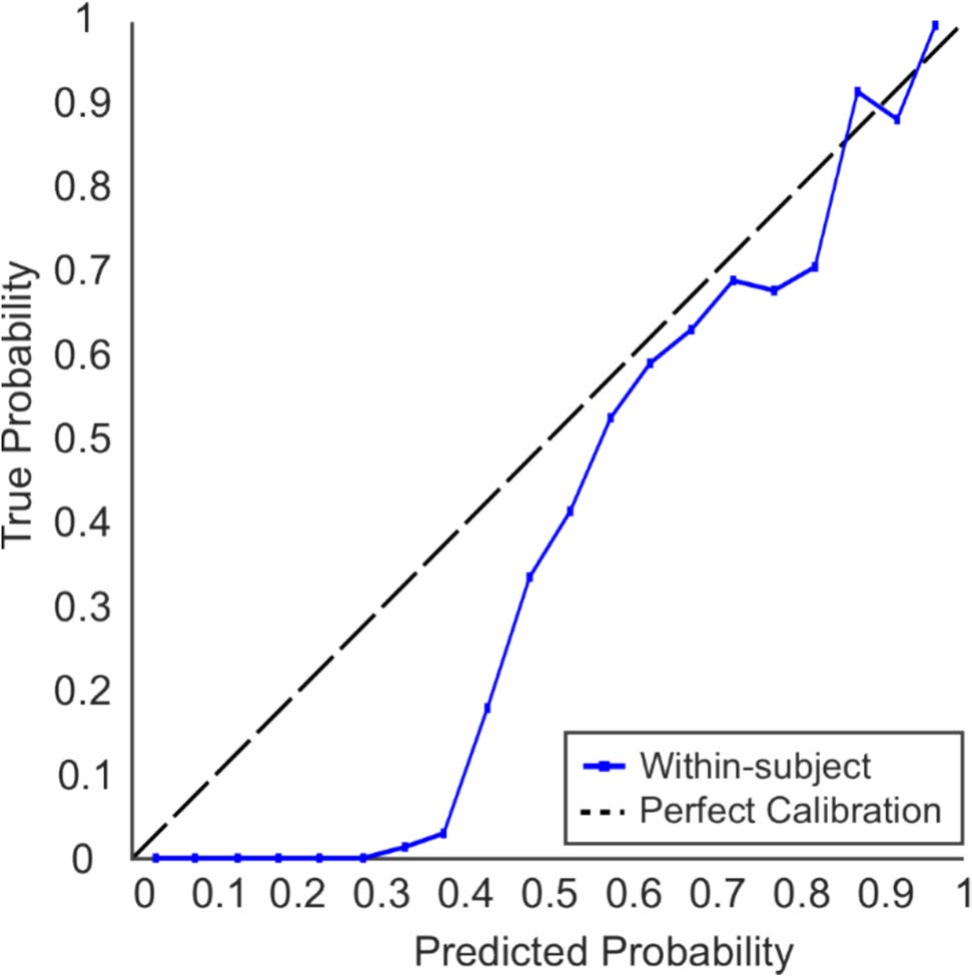
Calibration curve for the within-subject external validation sample for the scenes-faces classification task. The black dotted line represents perfect calibration

The calibration curves for the external validation set are presented in Fig. 4. To interpret the calibration curves, if the model line is above the perfect calibration line, it suggests that the model is underestimating the probability of the outcome. Whereas, in instances where the model line falls below the reference line, it suggests that the model is overestimating the probability of the outcome (Van Calster et al., 2019). The figure suggests that the model is not well calibrated. Whilst there is a reasonable trend towards the higher probabilities, the model tends to overestimate the probabilities of the outcome consistently, with greater overestimation occurring at the lower probabilities.

### Scenes: neutral – pain classification

The average model performance and optimal hyperparameters for the neutral and pain scenes are presented in Table 4. The RF model achieved an average cross-validation accuracy of 0.6120 ± 0.0220 and an AUC of 0.6659 ± 0.0358. For the within-subjects external validation sample, the model demonstrated an average accuracy of 0.5031 ± 0.0367 and an AUC of 0.5543 ± 0.061. A paired samples *t*-test demonstrated that model performance was significantly lower than the chance levels at the *p* = 0.05 threshold (t (26) = 10.21, *p* < .001, d = 1.964, 95% CI = [1.307, 2.609]). Regarding the individual subject performance, the model performance exceeded the chance threshold on two of the 27 subjects.

**Table 4:** Average sample performance metrics and hyperparameters for the neutral – pain scenes classification

	Cross-Validation		Within-Subject Validation	
Metric	Mean	SD	Mean	SD
Accuracy	0.6120	0.0220	0.5031	0.0367
AUC	0.6659	0.0358	0.5543	0.0612
Brier score	0.2249	0.0076	0.3600	0.0385
F1 score	0.6513	0.0320	0.6260	0.0591
Precision	0.5932	0.0224	0.4966	0.0304
Recall	0.7302	0.0873	0.8675	0.1536

**Optimal hyperparameters**: Number of estimators = 1840, Maximum depth = 21, Minimum samples to split = 14, Minimum samples at leaf = 4, Maximum features = sqrt, Bootstrap = False

### Faces: neutral – pain classification

The average model performance and model hyperparameters for the neutral and pain face classifications are reported in Table 5. The model demonstrated a cross-validated accuracy of 0.6660 ± 0.0459 and an AUC of 0.7315 ± 0.0588. For the within-subject external validation sample, the RF model achieved an average accuracy of 0.5284 ± 0.0395 and an AUC of 0.5506 ± 0.0668. A paired samples *t*-test demonstrated that the model performance was significantly lower than chance (t (26) = 5.97, *p* < .001, d = 1.150, 95% CI = [0.654, 1.632]). At the individual subject level, the model demonstrated accuracies that exceeded the chance level for three of 27 subjects.

**Table 5:** Average sample performance metrics and hyperparameters for the neutral – pain faces classification

	Cross Validation		Within-Subject Validation	
Metric	Mean	SD	Mean	SD
Accuracy	0.6660	0.0459	0.5284	0.0395
AUC	0.7315	0.0588	0.5506	0.0668
Brier score	0.2074	0.0167	0.2566	0.0143
F1 score	0.6132	0.0715	0.3205	0.1051
Precision	0.7159	0.0463	0.5677	0.1015
Recall	0.5402	0.0864	0.2360	0.0965

**Optimal hyperparameters**: Number of estimators = 1212, Maximum depth = 92, Minimum samples to split = 26, Minimum samples at leaf = 2, Maximum features = log2, Bootstrap = False

### Event-related potential

**Fig. 5:**
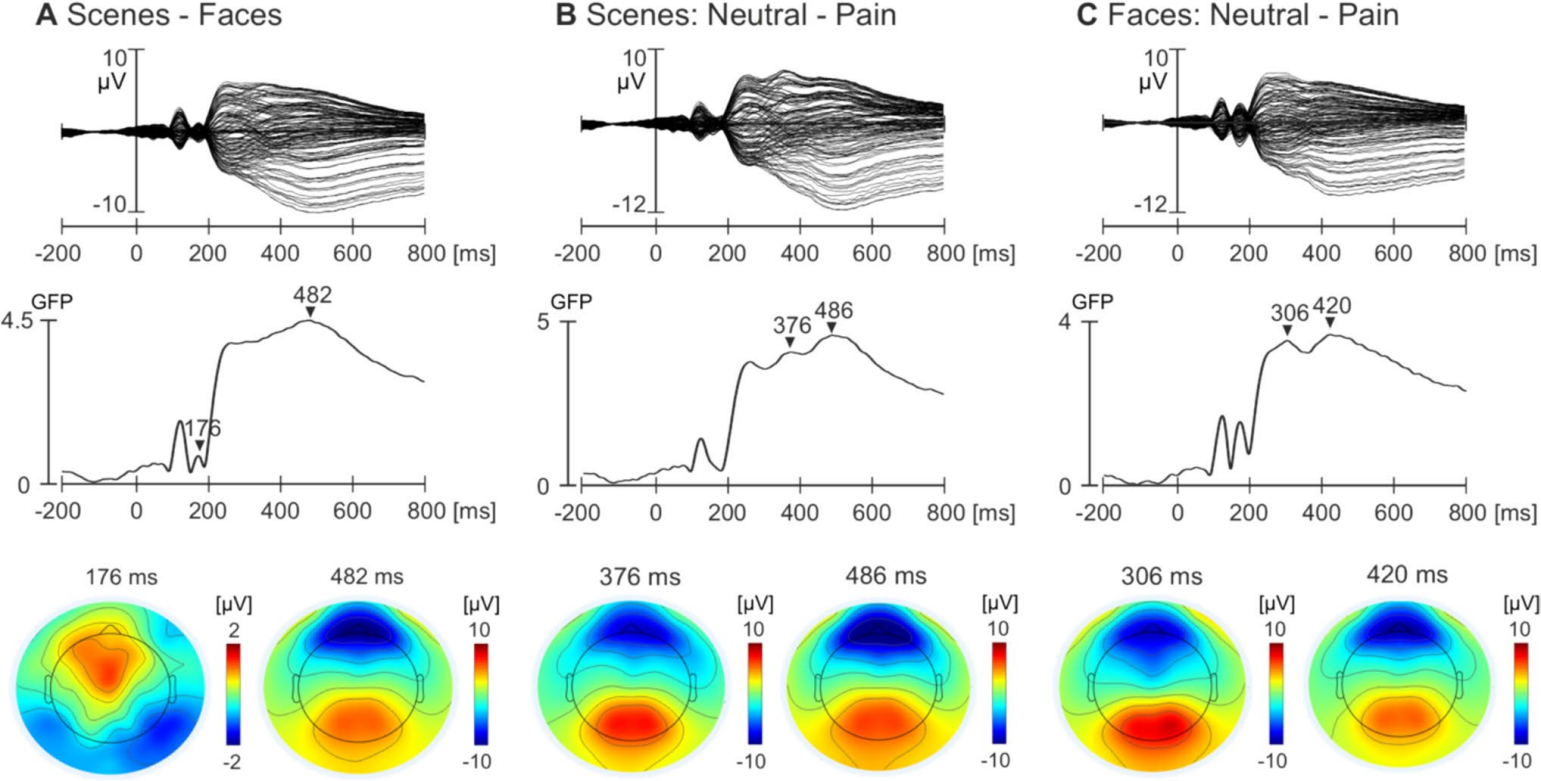
Grand average ERPs for the scenes – faces (**A**), neutral and pain scenes (**B**), and neutral and pain faces (**C**) comparisons. The top panel shows the butterfly plots, the middle panel shows the GFP, and the bottom panel shows the topographic maps for each of the three comparisons

Figure 5 shows the butterfly plots (top panel), global field power (GFP; middle panel), and topographic maps of the specific components identified (bottom panel) for the grand average of the two conditions for the scenes-faces (**A**), neutral and pain scenes (**B**), and neutral and pain faces (**C**) comparisons, respectively. For the scenes-faces comparison, a peak at 176 ms was identified, which exhibited negative potential in bilateral occipital-temporal electrodes and positive potential around the vertex region, corresponding to N170. An additional peak at 482 ms was identified in the time window of LPP. There was positive potential around central occipital electrodes, with strong negative potential over frontal electrodes. For the neutral and pain scenes comparison, a peak was identified at 376 ms (corresponding to P3), with a second at 486 ms (in the time window of LPP). Both components demonstrated strong positive potential over central occipital electrodes and strong negative potential over frontal electrodes. Finally, for the neutral and pain faces comparison, two peaks were also identified at 306 ms and 420 ms, corresponding to P3 and LPP components respectively. Similar to the scenes topography, a strong potential over central occipital electrodes and negative potential over frontal regions was evident.

**Fig. 6:**
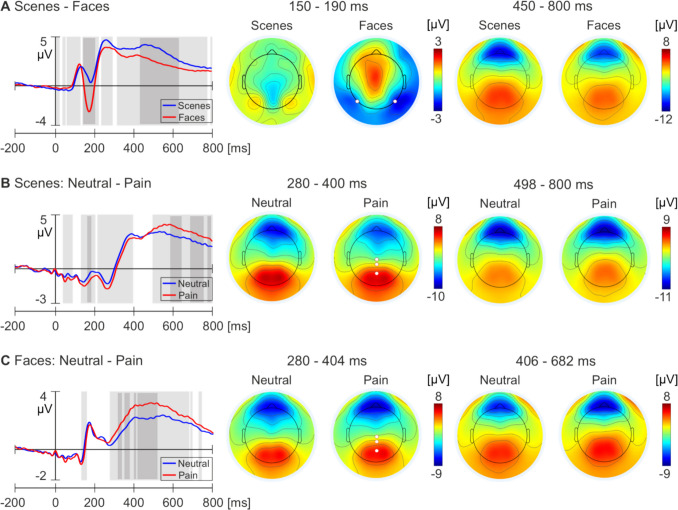
Average ERP waveforms and scalp isopotential maps for each comparison for all 62 subjects from the model development sample. **A** Brain responses during observation of face and scene images (regardless of pain component). Left panel: Average ERP waveforms from electrodes 58 (P7) and 96 (P8) for each condition. Middle panel: Average scalp potential for each condition between 150 and 190 ms. Right panel: Average scalp potential for each condition between 450 and 800 ms. **B** Brain responses during the observation of neutral and pain scenes. Left panel: Average ERP waveforms from electrodes Cz, 55, and 62 (Pz). Middle panel: Average scalp potential between 280 and 400 ms for each condition. Right panel: Average scalp potential between 498 and 800 ms for each condition. **C** Brain responses during the observation of neutral and pain faces. Left panel: average ERP waveforms at electrodes Cz, 55, and 62 (Pz). Middle panel: Average scalp potential between 280 and 404 ms for each condition. Right panel: Average scalp potential between 406 and 682 ms for each condition. The white circles denote the electrode locations of the averaged ERP waveforms. The light grey bars denote significant differences at *p* < 0.05. The dark grey bars represent significant differences at *p* < .00001

Figure 6 shows the average ERP waveforms from specific electrodes alongside the scalp isopotential maps for each condition and comparison. Regarding the faces – scenes comparison, a significantly stronger negative deflection during the observation of face images compared with scene images was recorded over bilateral occipital-temporal electrodes. The peak amplitude difference occurred using the N170 time window (146–204 ms; peak 174 ms; *p* < 0.00001). Moreover, the results demonstrated that scene images elicited greater amplitudes than the face images during the period of the LPP (316–772 ms; peak 462; *p* < 0.05). For the neutral and pain scenes images, the results demonstrated a difference in the LPP time window. A significantly stronger positive deflection was observed in a cluster of central-parietal electrodes (498–800 ms; peak 584 ms; *p* < 0.05) during the observation of pain scenes, which demonstrated greater amplitudes compared with the neutral condition. Regarding the neutral and pain face comparison, the results demonstrated that the pain face condition elicited greater amplitudes in a cluster of central-parietal electrodes during the P3 time window compared with the neutral face condition (280–404 ms; peak 404 ms; *p* < 0.05). Additionally, a similar pattern was observed during the LPP time window, with pain faces eliciting higher amplitudes than neutral faces (406–682 ms; peak 482 ms; *p* < 0.05).

## Discussion

We aimed to develop and externally validate a ML model for the classification of pain empathy by using single-trial EEG data. Initially, the present study assessed whether ML could classify discrete categories of visual stimuli, namely scene or face images, in line with our previous research (Mari et al., 2023c). The results demonstrated that the model could accurately discriminate between the picture categories, achieving accuracies that significantly exceeded chance performance. However, for both the neutral and pain scene and face image classification attempts, the results demonstrate that neither of the RF models developed exceeded chance performance on the external validation dataset, despite exhibiting promising cross-validation results. Taken together, the results support our first hypothesis that the RF model would accurately classify active observation of discrete categories of visual stimuli (scenes or faces) with accuracies exceeding chance levels on the external validation dataset. However, our second and third hypotheses, which predicted that the model would classify neutral and pain classes of scenes and faces with greater than chance levels, were not supported. The results suggest that active observation of vicarious pain stimuli could not be accurately classified using EEG and ML.

The findings from the current study are generally consistent with our previous research, which demonstrated the limits of the ML for the classification of visual stimuli and pain empathy (Mari et al., 2023c). In our previous work using a passive viewing paradigm, we demonstrated that ML could reasonably discriminate discrete categories of visual stimuli but failed to exceed chance performance for the vicarious pain classification (Mari et al., 2023c). Despite comparable performance for the faces and scenes classification, the results demonstrated a reduction in performance compared with our previous research, both in terms of model discrimination and calibration. Theoretically, increasing the training set sample size, and thus access to high-quality data, should have resulted in increased model performance (Rajput et al., 2023). However, for the faces and scenes classification, a reduction in performance, relative to the previous study, was observed.

The reduction in performance may also be the result of the increased sample size. Research has demonstrated that smaller samples can achieve seemingly high accuracies by chance, but as sample size increases, model performance tends towards chance-level performance (Combrisson & Jerbi, 2015). A review of prediction model studies from several clinical research domains, including Alzheimer’s, schizophrenia, autism, and psychosis, has demonstrated a negative association between sample size and model performance, with small samples tending to demonstrate greater model performance (Varoquaux, 2018). Indeed, our research lab has identified a similar trend in our attempts at pain intensity prediction, with model performance for EEG data significantly reducing as the sample size is increased (Mari et al., 2023a, b). Therefore, it is possible that the reduction in performance (for the scenes and faces classification) observed in the present study was influenced by the larger sample size. Therefore, the converging evidence across both studies is that the current “true accuracy” for ML analysis of EEG data for faces and scenes classification falls between 60% and 70%, which significantly exceeds chance performance. However, the observed performance still fails to exceed the level required for practical implementations. Research has suggested that to be clinically or practically relevant, models should achieve an AUC of at least 0.75 (Fan et al., 2006). Consequently, ML and EEG may prove an effective approach for the neural decoding of visual stimuli observation. However, to demonstrate true utility, improved model performance is imperative.

Our findings further reinforce the difficulty in classifying pain empathy using EEG data. Based on our previous study, it was predicted that implementing an active viewing paradigm would increase ERP amplitudes (Bennington & Polich, 1999), subsequently improving ML performance. However, the results showed no classification performance improvements across any of the three classification tasks relative to previous research from our lab (Mari et al., 2023c). It is possible that the active viewing paradigm did not increase the signal for the ERP waveforms to be sufficiently detected at the single-trial level. The ERP waveforms for both the neutral and pain conditions of either scenes or faces share highly similar spatiotemporal profiles, with differences between the conditions mainly implicated as enhanced or augmented component fluctuations (Coll, 2018; Fallon et al., 2015a). Whilst the results at the group level did show the expected pattern (e.g., altered P3, LPP; Coll, 2018; Mari et al., 2023c), it is possible that the patterns are not sufficiently different to detect at the single trial level. Consequently, we postulate that changing the experimental paradigm is not likely to have a significant impact on the ERP waveforms or classification performance.

Based on these findings, it is reasonable to argue that the low signal-to-noise ratio of EEG significantly hampers ML performance (Tivadar & Murray, 2019), but this phenomenon is most evident in external validation samples. This issue is likely exacerbated given the variability and noise issues associated with single-trial EEG analysis (Cohen & Cavanagh, 2011; Faisal et al., 2008; Kaplan et al., 2005; Marathe et al., 2014). As the neutral and pain classes have similar signal profiles (Coll, 2018; Fallon et al., 2015a; Mari et al., 2023c), the signal noise at the single-trial level likely prevents accurate classification. Moreover, given that ML models demonstrate increased performance when trained on high-quality datasets (Rajput et al., 2023), improving the signal quality will likely yield improved classification results. Therefore, attempts to develop ML and EEG empathy classification models should prioritise improving the signal-to-noise ratio.

Consequently, based on the current approach, it is possible that active viewing experiment paradigms do not significantly improve the classification performance of EEG-ML decoding tools. From a practical perspective, the utility of a brain decoding tool that requires active engagement to effectively classify responses is questionable. Such brain decoding tools are primarily desirable for individuals who cannot actively engage, such as pain assessment in individuals without the ability to self-report (Mari et al. 2022, 2023a) or brain-based speech decoding for individuals with communication impairments (Lopez-Bernal et al., 2022). Therefore, whilst improving model performance is imperative for practical utility, requiring a response would have significantly reduced the potential of the tool. Future attempts to improve the performance of an empathy classification algorithm are a necessity. Enhanced feature engineering and spatial filtering have been shown to increase the signal-to-noise ratio of EEG and ML performance (Blankertz et al., 2008; Rashid et al., 2020; Rivet et al., 2009; Singh & Krishnan, 2023). Moreover, alternative neuroimaging methods, which demonstrate improved signal quality, such as magnetoencephalography or electrocorticography, may enable improved classification results (Hill et al., 2012; Ploner & May, 2018; Schalk & Leuthardt, 2011; Simon et al., 2022; Singh, 2014). Whilst these approaches also suffer from limitations (e.g., increased expense and reduced practicality of magnetoencephalography) (Ploner & May, 2018; Singh, 2014), the potential for improved model performance necessitates exploration. Moreover, the results demonstrate a reduction in performance from the cross-validation to external validation stage for the faces and scenes classification, which suggests potential overfitting. Implementing regularisation techniques may help reduce the observed performance disparity and should be explored in future research. For example, research has demonstrated that regularised RF models outperform standard RF models (Deng & Runger, 2012; Mendez et al., 2021; Karthekeyan & Rooba, 2023). In addition, the results from the present study suggest that passive viewing paradigms should be conducted to maximise the utility of the approach, in the event of effective classification performance. Active viewing, based on the results of this study, appears to provide no benefit above passive efforts, whilst offering reduced utility and practicality.

The present study has several limitations. First, the scene and face images were not matched for all physical properties, which may have potentially confounded the EEG data. The physical properties of the images were consistent within image type, such as faces or scenes, but were not matched across conditions. Differing image properties, such as brightness, have been shown to alter the EEG response (Eroğlu et al., 2020). Moreover, given the inherent variability of the face and scene images, it is possible that the low-level visual properties of the image or eye movement variability (e.g., micro-saccades) could have contributed to the classification performance. Research has shown that stimulus properties could be decoded effectively by solely using eye movements during an active viewing task (Thielen et al., 2019). Moreover, it has been shown that eye movements can be effectively decoded from EEG data (Belkacem et al., 2015). Consequently, we cannot definitively rule out that the differing image properties or variability in eye movements contributed to the classification performance of the faces and scenes classification task. Future research should endeavour to ensure greater consistency across image types and explore the potential of eye tracking techniques. Second, we did not record the ethnicities of our sample, which also may have affected the results. Research has demonstrated that brain activation is attenuated when observing an individual from a different ethnicity to the observer (Cao et al., 2015). Third, the pain stimuli used in this study are artificial and may not be extreme enough to elicit EEG differences that enable classification at the single trial level. Future research may use stimuli that are more extreme (Osborn & Derbyshire, 2010) or that have increased ecological validity, such as videos, or virtual reality stimuli. For example, the recently proposed Empathy for Pain Stimuli System (Meng et al., 2023), which contains the Empathy for Action pain Video Database, which depicts 239 non-painful and painful videos of the whole body, may demonstrate increased realism over static photographs.

In addition, participants were required to consistently respond to the stimuli using either the left or right arrow keys. Because the keys were consistent for the pain and non-pain conditions, it is plausible that motor preparation could have confounded the classification of pain and non-pain conditions (Bracco et al., 2018). Future research should vary the response format to minimise the risk of motor preparation confounds. However, the effect of such systematic response confounds was not significant in the present study, as the ML model failed to classify pain and non-pain conditions during external validation. Additionally, in the present study, we only assessed model performance on a within-subject external validation dataset. Whilst within-subject assessment often demonstrates improved model performance (Mari et al., 2022; 2023b, c), cross-subject validation is arguably more important for brain decoding tools, because it does not require training on a specific individual to make accurate predictions (Tu et al., 2016). Therefore, future research should endeavour to assess ML performance on both within- and cross-subject validation sets to evaluate the true potential of the approach. Finally, the present study did not account for temporal drift in the neural signatures between the two experimental sessions. ERPs generally demonstrate reasonable test-retest reliability, especially when conducted within the same lab (Roach et al., 2020). However, measuring changes in neural dynamics would be beneficial as neural responses can significantly vary over time (Groen et al., 2022). Therefore, this study is limited by the lack of assessment of temporal changes in neural responses. Future research conducting within-subject temporal validation should assess the consistency of neural responses between sessions.

These findings provide several important contributions to the field. First, the current study provides support for our previous research that ML and EEG can successfully classify the observation of scenes and faces with performance significantly exceeding chance levels (Mari et al., 2023c). Our research demonstrates comparable performance across both passive and active viewing paradigms. However, this study also confirms the notion of the inability of ML algorithms to classify pain empathy from EEG data, which is in line with our previous findings (Mari et al., 2023c). To the best of our knowledge, we have conducted the only two pain empathy classification studies using EEG and ML and have demonstrated converging evidence. Furthermore, we identified that progressing from a passive to an active viewing paradigm did not improve model performance. Therefore, the poor classification results are not likely to be attributable to the task design but are more likely a result of technical factors such as the poor signal-to-noise ratio of EEG (Tivadar & Murray, 2019). Based on these results, we suggest that future attempts should prioritise improving the signal quality (e.g., improved processing or alternative neuroimaging approaches) in an attempt to obtain practically useful classification results (Fan et al., 2006). Moreover, our findings further reiterate the importance of external validation. External validation is the process of evaluating model performance on an external, novel dataset, collected at a different time, geographical location, or using a different experimental paradigm (Moons et al., 2015). Without external validation, from both this study and the previous research, it could be concluded that ML and EEG can accurately classify pain empathy based on cross-validation results (Mari et al., 2023c). However, the external validation results demonstrate that performance does not generalise to new data. Models that are only internally validated provide overoptimistic results, which are likely affected by overfitting (Cabitza et al., 2021; Siontis et al., 2015; Vabalas et al., 2019). Despite external validation being an imperative aspect of ML, it is seldom conducted (Cabitza et al., 2021; Mari et al., 2022; Moons et al., 2015; Ramspek et al., 2021). Consequently, most of the ML research published to date is uninterpretable, as overfitting cannot be discounted. To prevent the onset of a new replication crisis, external validation and transparent reporting are essential (Hutson, 2018).

This study demonstrates the capability and potential limits of ML for pain empathy classification using EEG data. Initially, we demonstrated that the classification of discrete visual stimuli (faces and scenes) could be reasonably decoded by using ML and EEG, replicating our previous research. We also demonstrated that ML and EEG remain ineffective for classifying pain empathy, despite our attempts to enhance the differences between the two classes using an active observation paradigm. Therefore, this study suggests that active viewing is not sufficiently advantageous to ML performance. The current study demonstrates both the potential and limitations of ML for decoding brain states, again providing robust estimates with large sample sizes and thorough external validation procedures that are too often lacking in this research area.

## Data Availability

The EEG data is available freely through the Open Science Framework (https://osf.io/2e8zk/). Materials for the experiments are available upon reasonable request, and none of the experiments were preregistered.

## Code availability

The code for the analysis is available upon reasonable request.
